# Roles of Plasmalemma Aquaporin Gene *StPIP1* in Enhancing Drought Tolerance in Potato

**DOI:** 10.3389/fpls.2017.00616

**Published:** 2017-04-25

**Authors:** Li Wang, Yuhui Liu, Shoujiang Feng, Jiangwei Yang, Dan Li, Junlian Zhang

**Affiliations:** ^1^Gansu Key Laboratory of Crop Genetic and Germplasm Enhancement, Gansu Provincial Key Laboratory of Aridland Crop Science, Gansu Agricultural UniversityLanzhou, China; ^2^Department of Plant Biotechnology, College of Life Science and Technology, Gansu Agricultural UniversityLanzhou, China; ^3^Institute of Soil, Fertilizer and Water-saving Agriculture, Gansu Academy of Agricultural SciencesLanzhou, China; ^4^Department of Agronomy, Longdong UniversityLanzhou, China; ^5^Department of Olericulture, College of Horticulture, Gansu Agricultural UniversityLanzhou, China

**Keywords:** aquaporin, carbon starvation, drought tolerance, potato, nonstructural carbohydrates

## Abstract

Survival and mortality of plants in response to severe drought may be related to carbon starvation, but little is known about how plasma membrane intrinsic proteins may help alleviate the drought-induced damage. Here, we determined the roles of plasmalemma aquaporin gene in improving plant water status, maintaining carbon accumulation, and thereby enhancing drought tolerance. Seven *StPIP1* transformed potato (*Solanum tuberosum* L.) lines (namely T1, T2…T7) were compared with non-transgenic control plant at molecule and whole-plant levels. The relative expression of *StPIP1* gene was found in leaves, stems and roots, with the most abundant expression being in the roots. The transgenic lines T6 and T7 had the highest *StPIP1* expression, averaging 7.2 times that of the control and the greatest differences occurred 48 h after mannitol osmotic stress treatment. Using an evaluation index to quantifying the degree of drought tolerance, we found that the *StPIP1* transgenic lines T6 and T7 had the highest drought tolerance, averaging 8.5 times that of the control. Measured at 30 days in drought stress treatment, the control plant decreased net photosynthetic rate by 33 and 56%, respectively, under moderate and severe stresses; also decreased stomatal conductance by 39 and 65%; and lowered transpiration rate by 49 and 69%, compared to the no-stress treatment, whereas the transgenic lines T6 and T7 maintained a relatively stable level with slight decreases in these properties. The constitutive overexpression of *StPIP1* in potato improved plant water use efficiency and increased nonstructural carbohydrate concentration, which helped alleviate carbon starvation and minimized the loss of biomass and tuber yield due to drought stress. We conclude that the expression of *StPIPs* improves overall water relations in the plant and helps maintain photosynthesis and stomatal conductance; these help minimize carbon starvation and enhance the whole plant tolerance to drought stress.

## Introduction

The shortage of fresh water is one of the most severe constraints in crop productivity in arid and semiarid areas (Toenniessen et al., 2003; Chai et al., 2016). Climate change may increase number of extreme weather events in the future (IPCC, 2014), which will undoubtedly increase stresses to plants (Smirnov et al., 2016; Stott, 2016). Genetic enhancement of plant tolerance to drought will play a key role in combating the abiotic stress (Norton et al., 2016). Plant resistance to drought relies heavily on the maintenance of the osmotic and ionic homeostasis by means of the transportation of water and metabolic activities by membranes (Chen and Polle, 2010; Ruiz-Lozano et al., 2012).

Aquaporins (AQPs) are integral membrane proteins that serve as channels in the transfer of water, and in some cases, small solutes across the membrane. These proteins respond to water availability in a timely manner (Ding et al., 2015) and the exploration of the AQP responses to drought will help improve plant water use efficiency (Hu et al., 2012; Moshelion et al., 2015). AQPs belong to a large family of major intrinsic proteins (MIPs), and are categorized into four clades, namely plasma membrane intrinsic proteins (PIPs), tonoplast intrinsic proteins, small basic intrinsic proteins, and nodulin 26-like intrinsic proteins (Baiges et al., 2002). All four types of AQPs display highly conserved structures wherein six membrane-spanning alpha helices are linked by five short loops with their N- and C-termini toward the cytosol. Two of these loops contain highly conserved asparagine-proline-alanine motifs, which play an important role in the formation of water-selective channels (Chaumont et al., 2001). Drought stress often leads to an altered water homoeostasis within the plant, and in response, AQPs provide timely signaling to transport water among plant organs (Vera-Estrella et al., 2004). Therefore, the efficiency of water transport in plant tissues relies on plasma membrane intrinsic proteins (Zhou et al., 2007).

The capacity of drought tolerance varies with plant genetics and physiological characteristics. The AQPs are responsible not only for the transport of water and some solutes across cell membranes (Soto et al., 2012), but also CO_2_ exchange in some plant species like tobacco (*Nicotiana tabacum* L.) (Uehlein et al., 2003). Additionally, AQPs play a critical role in plant regeneration, cell elongation, stomatal opening, fruit ripening and seed germination (Forrest and Bhave, 2007). In many plant species, AQPs provide a positive effect on abiotic and biotic stresses. In banana (*Musa acuminate* L.), the transgenic lines overexpressing *MusaPIP1;2* have a better ability to recover from abiotic stress (drought, salt, and cold) than non-transgenic lines (Sreedharan et al., 2013). In rice (*Oryza sativa* L.), *OsPIP1-1* and *OsPIP2-1* genes play a positive role in recovery from chilling stress (Yu et al., 2006). In pepper (*Capsicum annuum* L.), silenced *CaPIP1-1* inhibits plant growth and decreases tolerance to salt stress (Yin et al., 2015). In transgenic tobacco, PIP1b is found to enhance plant vitality under certain conditions, but it influences plant growth negatively (Aharon, 2003). In these studies, the gene expression and physiological characterization of whole plant are often considered the main responses to abiotic stresses. Little has been reported how *PIPs* genes may influence plant water status and carbon starvation, as the latter was considered an important mechanism of plant survival to drought (McDowell et al., 2008). An unanswered question is that: do *PIPs* genes directly affect carbon starvation and thus enhancing plant tolerance to drought stress?

Potato (*Solanum tuberosum* L.) account for one third of the primary crop production in the world (Faostat, 2014), and it plays a significant role in securing food supply on the planet (Gerbens-Leenes et al., 2009). Potato yield is highly related to the host plant genetics and crop management practices (El-Shraiy and Hegazi, 2010; Khan et al., 2016). Water deficit during the reproductive period decreases potato carbon accumulation (Obidiegwu et al., 2015), reduces carbon remobilization from vegetative tissues to tubers (Ojala et al., 1990), and decreases the number of tubers and tuber size (Cavagnaro et al., 1971). One of the promising options to alleviate those water deficit-induced consequences is to use transgenic plants (Qaim and Kouser, 2013). Many genes through genetic engineering have been found to help enhance drought tolerance in potato (Mittler and Blumwald, 2010). However, little has been reported how AQPs, the prominent proteins involved in the maintenance of water homoeostasis in plants, would function in combating water deficit in potato.

A novel plasma membrane AQP gene- *StPIP1* was identified in potato (Wu et al., 2009) and the gene has been successfully transformed in the reverse orientation into model tobacco through *Cauliflower mosaic virus* (CaMV) 35S promoter. However, in the transgenic tobacco plants, the expression of *StPIP1* mRNA was decreased substantially under drought stress (Wu et al., 2009). The decreased *StPIP1* expression was reflected in the delay of seed germination, abnormal leaf elongation, slow seedling growth, and accelerated seedling wilt. In contrast, *StPIP1* was found to play an important role for water transportation in potato (Wu et al., 2009), as *StPIP1* protein shares a high homology with several other PIPs, such as NtAQP1, PhPIP, and CaPIP1, found in other Solanaceae plants (Yin et al., 2015). Also, a body of evidence shows that *StPIP1* gene may have a great potential for developing drought-tolerant potato. Following the previous work, we transferred *StPIP1* gene to a most abundantly-grown potato cultivar- “Shepody” in an process using the constitutive promoter CaMV 35S. We hypothesize that (i) the *StPIP1*-transformed potato plants have an enhanced tolerance to severe drought compared to non-transgenic potato, and (ii) the overexpression of *StPIP1* gene helps maintain plant water status and alleviate carbon starvation. To test the hypotheses, we conducted a series of comprehensive investigations, including (i) determining the whole-plant biophysicochemical traits, such as stomatal conductance, transpiration regulation, photosynthesis efficiency, nonstructural carbohydrates (NSC) contents, and water use efficiency (WUE); (ii) quantifying the degree of the tolerance using evaluation indices; and (iii) assessing plant performance and productivity, such as tuber yield under field conditions. These measurements support the hypothesis that the constitutive expression of *StPIP1* gene helps maintain plant water status and alleviates the whole-plant carbon starvation under drought stress.

## Materials and methods

### Plant materials and growth conditions

The experiment was carried out at Gansu Agricultural University in 2012 and 2013. The potato tetraploid cultivar “Shepody” was used in all tests. The plants were propagated *in vitro* by subculturing single-node cuttings on MS liquid medium supplemented with 3% (w/v) sucrose and grown under light intensity of 60–80 μmol m^−2^ s^−1^ and a photoperiod of 8 h d^−1^ at 22 ± 1°C. After culturing for 25 d, MS liquid medium was removed and the microtuber inducing medium was added with the supplement of 8% (w/v) sucrose and 5 mg L^−1^ of 6-BA. Plantlets were grown under dark at 22 ± 1°C and microtubers were harvested from the 8-week-old plants.

### Plasmid construction

The 867 bp cDNA of the *StPIP1* gene (DQ999080) isolated from potato cultivar “Gannongshu No.2” was cloned into the *Bam*H I –*Sac* I site of the plasmid pStPIP1 containing CaMV 35S promoter (Wu et al., 2009), such that the β*-glucuronidase* (*GUS*) gene was replaced by *StPIP1* gene. The recombinant plasmid pStPIP1 was introduced into *Agrobacterium tumefaciens* strain *LBA4404* using the freeze-thaw method (Höfgen and Willmitzer, 1988). The presence of the plasmid was verified by restriction enzyme digestion and PCR amplification.

### Potato transformation, PCR and PCR-southern analysis

Slices of microtubers of the potato cultivar “Shepody” were used as the receptor for *Agrobacterium*-mediated transformation, which was performed following the procedures described elsewhere (Si et al., 2003). The regenerated shoots of 10.0 mm in height were transferred to the selective rooting medium (MS+100 mg L^−1^ Kanamycin+200 mg L^−1^ Carbenicillin). Genomic DNA was isolated from 0.1 g of leaves from putative transgenic and non-transgenic control plants using the cetyl-trimethyl ammonium bromide (CTAB) method (Edwards et al., 1991). The presence of transferred neomycin phosphotransferase II (*Npt II*) gene was confirmed using standard PCR technique. The *Npt II* gene was amplified with the forward primer 5′-GCTATGACTGGGCACAACAG-3 ′ (1–18 bp) and the reverse primer 5′-ATACCGTAAAGCACGAGGAA-3′(657–676 bp), which generated an expected PCR product fragment of 676 bp. Amplification was performed in a thermal cycle (UNO II, Biometra) programmed for one cycle of 3 min at 94°C, followed by 30 cycles of 45 s at 94°C, 45 s at 59°C, and 1 min at 72°C. A final extension step was performed for 5 min at 72°C. The PCR products were electrophoresed on a 0.8% agarose gel, denatured, and transferred to a positively charged nylon membrane (Roche). Membranes were hybridized at high stringency in hybridization buffer at 42°C for 16 h. Membranes were washed twice in 2 × SSC, 0.1% (v/v) SDS at room temperature for 15 min each, and twice in 0.5 × SSC, 0.1% (v/v) SDS at 65°C for 15 min each. The gene-specific probe of the *Npt II* gene was labeled using a DIG High Prime DNA Labeling and Detection Starter Kit I (Roche). The hybridization signal generated using NBT/BCIP color substrate was detected after 20 h.

### Southern blot analysis

Genomic DNA was extracted using the CTAB method (Edwards et al., 1991). Forty micrograms of genomic DNA were digested with the restriction endonuclease *Hin*dIII and separated on 0.8% agarose gel, denatured, and transferred to a positively charged nylon membrane (Roche). Membranes hybridization and detection methods were the same as those used in the PCR-Southern analysis described above.

### Reverse transcription PCR assay (RT-PCR) analysis

Total RNAs were isolated using the RNA simple Total RNA Kit (TIANGEN, lot#N2822) following the manufacturer's instructions. Reverse transcription was performed in 20 μL reaction mixtures using the PrimeScript™ RT reagent Kit with gDNA Eraser (TaKaRa, RR047A). The expressed *Npt II* gene was confirmed using standard PCR techniques. The *Npt II* gene primers and amplification methods were the same as those in the PCR analysis described above. The RT-PCR products were electrophoresed on a 0.8% agarose gel.

### Mannitol osmotic stress treatments

Seven transgenic potato lines (designated as T1, T2, …T7) and the control (a non-transgenic potato cultivar, NT) were included in the test. For plant mannitol osmotic abiotic stress treatments, the six-leaf-stage T7 and the non-transgenic (NT) plants were grown in liquid MS medium containing 3% (w/v) sucrose, and then the liquid medium was removed and replaced by 50 mL of liquid MS medium containing 3% (w/v) sucrose and 25 mM mannitol. Plant leaves were collected at 0, 6, 12, 24, 48, and 96 h after stress treatment, respectively. The entire experiment was run three times. The samples were cleaned with sterilized water, immediately frozen in liquid nitrogen, and stored at −80°C for subsequent analysis.

### Gene expression assays with quantitative real-time PCR

Total RNA was extracted from mannitol-treated samples of T7 and non-transgenic (the control) potato plantlets using the RNA simple Total RNA Kit (TIANGEN, lot#N2822). The RNA was quality checked and quantified using a Nanodrop ND-1000 (Nanodrop Technologies, USA). Reverse transcription was performed in 20 μL reaction mixtures using the PrimeScript™ RT reagent Kit with gDNA Eraser (TaKaRa, RR047A), and qRT-PCR amplification was conducted in 20 μL reaction mixtures with the SYBR® Premix Ex Taq™ II(Tli RnaseH Plus) (TaKaRa, DRR820A), and 10 μM of each primer (*ef1a* as internal control gene and forward and reverse primers: 5′-CAA GGATGA CCC AGC CAA G -3′ and 5′-TTCCTT ACC TGAACGCCT GT-3′; *StPIP1* gene-specific forward and reverse primers: 5′-TGTGGG TAT TCA AGGAGTTGC T-3′and 5′ -CCA AGA ACA GAC CAA ATG TCA C-3′). Reactions were conducted on a Mxprosystem (Applied Stratagene Mx3005p real-time PCR) using the default cycling conditions (30 s at 95°C and 40 cycles of 5 s at 95°C, and 34 s at 60°C). The transcripts of stress-responsive genes are shown as relative transcripts compared with NT under non-stress growth conditions. Each Quantitative Real-Time PCR was repeated three times independently. A blank control was included where no cDNA was added in the reaction mixtures. After each reaction, melt curve analysis was used to verify the specificity of amplification and the relative expression levels calculated by 2^−ΔΔCt^.

### Physiochemical assessments of transgenic potato carrying *StPIP1* gene

Mini-tubers of the transgenic potato lines carrying the *StPIP1* gene were evaluated for their stress tolerance in controlled-environment greenhouses. Potato mini-tubers were produced from test-tube plantlets following the established procedures (Li et al., 2012). One mini-tuber recovered from dormancy was seeded in each pot (25 cm in height and 32 cm in diameter) filled with 15% vermiculite (v/v) and 85% loessal soil under room temperatures. A daily watering was provided to each pot to maintain soil moisture at 70–80% field capacity. Healthy plants with a similar physical size were selected for drought treatments and those plants that survived the drought challenge were measured further.

All seven transgenic lines and the non-transgenic check were tested under three water treatments: 80–90% field water holding capacity (FC) as non-stress treatment, 55–60% FC as moderate drought, and 30–35% FC as severe drought. The three water stress treatments were implemented by measuring soil moisture at 10:00 and 16:00 h each day using moisture meters TDR-300 (Spectrum®, USA) that were pre-installed in each pot, and an amount of water was, respectively added to each plot to bring the soil water content to the pre-designed level.

The treatments were arranged in a completely randomized design with three replicates. Each treatment in each replicate had 10 pots, one plant per pot, with a total of 720 pots (8 lines × 3 stress-treatments × 3 replicates × 10 pots each). The sufficient number of plants allowed all measurements and analyses to be taken below. The day/night temperatures in the greenhouse were set at 25/15°C and relative humidity at 70 to 75%. The day/night photoperiod was 14/10 h. Light intensity was 3500 lx. At Day 30 after drought treatments, plant heights were measured from the soil level to the tip of the main stem, the leaf size was measured with the weighted estimate of paper outline, and the stem thickness were measured using a vernier caliper. The relative expansion rate of leaf area was expressed as: leaf area expansion rate = (the average leaf area at Day 30 after drought treatment–the average leaf area before the treatment)/30. Daily gain of plant height = (the average plant height at Day 30 after drought treatment−the average plant height before the treatment)/30. Stem thickness expansion rate = (the average stem thickness at Day 30 after drought treatment−the average stem thickness before the treatment)/30. A portable photosynthetic system LI-6400XT (Li-COR, Lincoln, NE, USA) was used to estimate PN, Tr, Gs and Ci for the second terminal leaflet of fully grown second leaf at an irradiance of 1500 μmol m^−2^ s^−1^ and CO_2_ concentration of 400 μmol mol^−1^. The fifth or sixth leaves below the growing point were selected for the determination of soluble sugar, starch, nonstructural carbohydrates (NSC), total chlorophyll content, proline content, relative water content (RWC), malondialdehyde (MDA), electrolyte leakage, superoxide dismutase (SOD), catalase (CAT) and peroxidase (POD). The soluble sugar, starch and NSC were measured using the method described by Yu (Yu et al., 2011). The electrolyte leakage and RWC content was determined with the method of Yu (Yu et al., 2006). The total chlorophyll content, proline content, MDA, SOD activities, POD activities and CAT activities were determined according to the method described by Li (2000). The survival rate was calculated at Day 65 after drought treatments and expressed as the ratio of number of plants survived drought treatments to total number of plants before the drought treatments.

### Biomass, yield, and root activity in transgenic potato carrying *StPIP1* gene

All plants were hand-harvested at the full maturity, about 90 days after seedling emergence. For each plant, shoot, roots, and tubers were weighed separately for fresh wrights, and then the samples were oven-dried at 70°C to a constant weight and weighed for the dry weights of shoot, roots, and tubers. The entire experiment was run three times, using three planting dates April through August in 2013.

In addition to the plant biomass and tuber yield, we determined “root activity” of the plants under the different levels of drought stress. Root activity is considered one of the important traits that reflect root respiration, oxidizing activity, or reducing activity of plants in response to stress. Measurement of root activity gives an indication of how plant roots perform under stress (Muler et al., 2014; Whalley et al., 2017; Wu et al., 2017). For instance, where the nutrient absorption by roots is inhibited due to drought stress, the oxidizing activity of the roots is low (Muler et al., 2014). A number of methods can be used to measure root activity. In the present study, we used the triphenyl tetrazolium chloride (TTC) method (Zhang et al., 2015a). In brief, plant roots were washed with deionized water and excised at 5 cm in length from the root tips on the 30th day after stress treatment. The reaction mixture consisted of 5 g samples of root tips, 5 ml of 0.4% TTC, and 5 ml of phosphate buffer solution (pH 7.0) in a beaker, with root tips fully immersed in the solution for 1 h at 37°C in the dark. When root tips turned red in color, 2 mL of 1 M sulfuric acid was added to stop the reaction. Root tips were dried with filter paper and homogenized with liquid nitrogen in an ice cold mortar and pestle. The red extraction was moved into a centrifuge tube to make the total volume of 10 ml using ethyl acetate. The extraction was vortexed for 30 s, and centrifuged at 1000 rpm for 5 min. The extraction was measured at 485 nm against the blank of ethyl acetate. The dehydrogenase activity is regarded as an indicator of the root activity. This analysis was repeated for three times.

### Genetic stability analysis

Genomic DNA was isolated from 0.1 g of leaves from the putative transgenic and the NT control plants. The *Npt II* gene was demonstrated using standard PCR techniques. The genomic DNA isolation methods, the *Npt II* gene' primers and the PCR reaction programs were the same as those described in the above sections. The PCR products were electrophoresed on a 0.8% agarose gel.

### Quantification of drought tolerance

To quantify the degree of drought tolerance, we employed an integrated evaluation index where a cluster analysis was combined with standard deviation coefficient allocation weighted method. Detailed methodology was described previously (Wang et al., 2013). In brief, an evaluation index was developed using the following three steps:

- Data standardization, where a subordinator function was formed to standardize the measured raw data of each parameter as follows:
  (1) $$\begin{array}{r} \mu \left(X_{ij}\right)=\frac{\left(Xij-X\min\right)}{\left(X\max-X\min\right)} \end{array}$$
  (2) $$\begin{array}{r} \mu \left(X_{ij}\right)=\left(\frac{X\max-Xij}{X\max-X\min}\right), \end{array}$$
  where, *Xij* is the value of the *j*th variable (daily gain of plant height, leaf area expansion rate, stem thickness expansion rate, plant tissue water contents, tuber yield per plant, and rate of plant survival to drought treatment.) of the *i*th genotype, Xmin and Xmax represent the minimal or maximal value among the X*ij* values for the *j*th variable, respectively. The subordinator function with a positive correlation between a particular growth variable and drought tolerance was expressed by Equation (1), while a negative correlation was expressed by Equation (2).
- Allocation of weights: Equation (3) below was used to calculate the standard deviation coefficient *Vj* while Equation (4) was used to summarize the weighted coefficient *Wj* of specific variables.
  (3) $$\begin{array}{r} Vj=\sqrt{\frac{\overset{n}{\underset{i=1}{\sum }}{\left(Xij-\bar{Xj}\right)}^{2}}{\bar{Xj}}} \end{array}$$
  (4) $$\begin{array}{r} Wj=\frac{Vj}{\overset{m}{\underset{j=1}{\sum }}Vj}, \end{array}$$
  where, *n* represents the number of genotypes (seven transgenic lines plus the non-transgenic check) used for drought challenges, *m* represents the six plant-growth traits: daily gain of plant height, leaf area expansion rate, stem thickness expansion rate, plant tissue water contents, tuber yield per plant, and rate of plant survival.
- An integrated evaluation index: Equation (5) below was used to generate an integrated evaluation index as follows:
  (5) $$\begin{array}{r} D=\overset{m}{\underset{j=1}{\sum }}\left[\mu \left(Xij\right)\times Wj\right]\;j=1,2,\ldots ,m \end{array}$$
  where, D is the integrated evaluation index, *Xij* is the *j*th parameter in the *i*th line of the matrix, *Wj* is the weighted coefficient of a specific parameter, *n* represents the seven transgenic lines used for stress challenges as well as the control, and *m* represents the six key plant-growth traits that are integrated. With this integrated evaluation index, the response of potato genotypic lines to the different levels of drought stress was evaluated in a quantitative manner.

### Statistical analysis

Preliminary analysis showed that the treatment effects followed a similar trend in the first, the second, and the third runs of the experiment, and there were no significant treatment by run interactions for most of the variables measured. Therefore, the data from the three runs were pooled together in the analysis. All data were subjected to Tukey's HSD test using the SPSS package (SPSS Software, 19.0, SPSS Institute Inc., USA). Cluster analysis and the quantitative indices were used to categorize the seven genetic lines into the different levels of drought tolerance: very high, high, low, and very low. Significances between treatments in the ANOVA or among the categories of drought tolerance were considered at *P* < 0.05.

## Results

### Plasmid construction and potato transformation

The plasmid pStPIP1 containing the *StPIP1* gene was constructed and both *StPIP1* and *Npt II* genes were verified with PCR amplification (Figure S1). Green shoots were produced directly from the transformed micro-tuber slices after 4 to 5 weeks of culture in the selective medium (Figure S2). Roots were formed about 5 days after green shoots were transferred to the selective rooting medium (Figure S3). These growth traits did not appear in the non-transgenic control lines. Potato plantlets with well-developed roots were propagated for further molecular analyses as follows.

### Molecular detection and gene expression analysis

**Figure 1:**
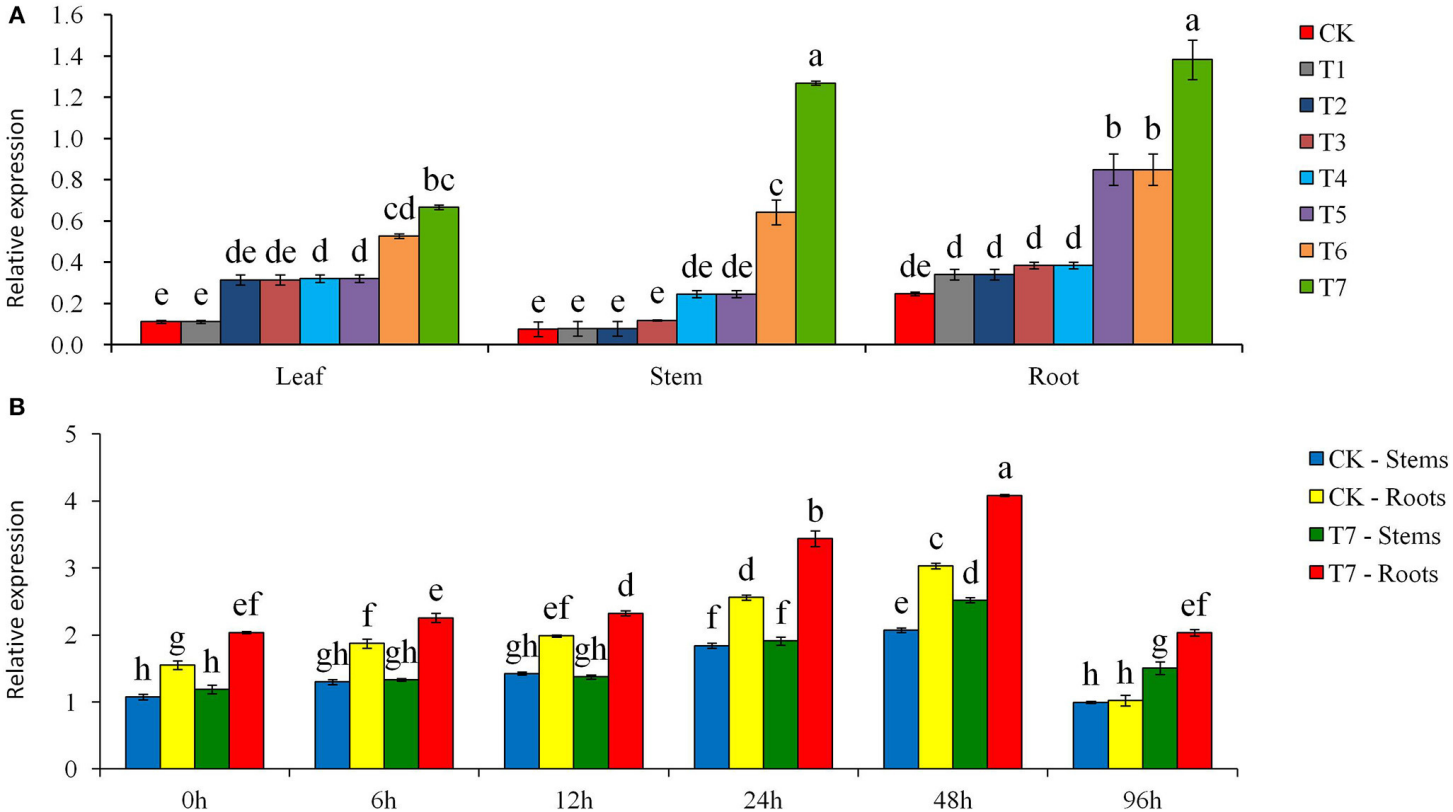
**Expression of ***StPIP1*** gene with quantitative real-time PCR. (A)** Assay of tissue-specific expression of *StPIP1* gene in transgenic (T1 to T7) and non-transgenic (CK) potato plants, and T7 root was normalization factor, **(B)** Effects of mannitol-induced osmotic stress treatments on the relative quantity of expression levels of the *StPIP1* gene in the transgenic line T7 (as an example) and non-transgenic control potato plants, and CK stem in 0 h stress was normalization factor. Significant differences among means over the period (h) of mannitol stress treatments were determined according to Tukey's HSD test at *P* < 0.05. The line bars are standard errors (*n* = 9, i.e., 3 replicates × 3 runs of the experiment).

PCR analysis using *Npt II* gene-specific primers showed that the putatively transformed plants had an amplification product of 676 bp and this product was missing in the control plant (Figure S4). The PCR amplification results were further confirmed by PCR-Southern blot analysis, which showed that the amplification product of 676 bp was the *Npt II* gene where 9 transformed plants had the *Npt II* gene and the control plants did not (Figure S4B). The Southern blot analysis revealed that *Npt II* gene was integrated into the potato genome, with the 7 transgenic plant lines having the hybridization signal, of which the line T4 having two copies of *Npt II* gene (Figure S5). The expression profiles for *Npt II* mRNA in the leaves of the transgenic plants showed that the *Npt II* gene was expressed and no transcript was found in the control plants (Figure S6). The qRT-PCR analysis showed that *StPIP1* was expressed in all plant tissues with higher expressions in the stems and roots than in the leaves (Figure 1A). In each tissue, the relative expression of the *StPIP1* gene was significantly higher in transgenic plants than in the control plants. Also, there were significant differences among the transgenic lines; T6 and T7 lines had the greatest *StPIP1* expression, which were 2–15 times that of the four other transgenic lines, and 5–15 times that of the control plant (Figure 1A). In both transgenic and control plantlets, the expression of the *StPIP1* gene was enhanced with the time of osmotic treatment until 96 h (Figure 1B). At a given time, the relative expression of *StPIP1* gene was highest in the T7 lines.

### Genetic stability

The PCR amplifications of *Npt II* proved that six (T1, T2, T4, T5, T6, and T7) of the seven transgenic lines carried the complete coding sequence of *Npt II* gene in 676 bp, and no specific fragment was detected in the control or in the T3 line (Figure S7). These results confirmed the genomic integration and stabilized inheritance of the *Npt II* gene carried by the majority of transgenic potato lines. Thus, the six transgenic lines (excluding T3) were carried in the evaluation of drought tolerance for over-expression of *StPIP1* gene.

### Evaluation of drought tolerance for overexpression of *StPIP1* gene

The following three steps were taken for the assessment of plant tolerance to drought. First, the six key plant growth traits were individually included in the evaluation formula: (i) daily gain of plant height, (ii) leaf area expansion rate, (iii) stem thickness expansion rate, (iv) plant tissue water contents, (v) tuber yield per plant, and (vi) rate of plant survival to drought treatment. Evaluated using the six individual growth traits, the control plants had a significantly (*P* < 0.05) lower drought tolerance than all the transgenic lines, reflected by lower coefficients (Table 1). There were significant differences in the degree of drought tolerance among the transgenic lines. The line T7 had a significantly higher coefficient of drought tolerance with higher water content (95%) than the T6 line, while T7 had a lower coefficient to tuber yield than T6. Both lines had an equivalent value on leaf area expansion and survival rate. Thus, using the individual growth traits to rank the relative drought tolerance among plants was complicated because each growth trait had a different degree of influence on the magnitude of drought tolerance.

**Table 1:** **Coefficients of drought tolerance evaluated for each of the six main plant growth variables for the transgenic potato lines overexpression of ***StPIP1*** gene and the check cultivar**.

**Genotype^a^**	**Daily gain in height (%)**	**Leaf area expansion rate (%)**	**Stem thickness expansion rate (%)**	**Relative water content (%)**	**Yield per plant (%)**	**Survival rate (%)**
CK	48.2 ± 1.1 c	45.3 ± 5.3 b	53.1 ± 0.9 c	73.1 ± 1.5 c	32.4 ± 0.5 f	58.3 ± 1.0 c
T7	80.4 ± 0.9 a	73.6 ± 3.4 ab	75.2 ± 0.5 ab	95.1 ± 0.7 a	74.3 ± 0.3 b	84.4 ± 1.8 ab
T6	80.9 ± 1.0 a	90.0 ± 6.2 a	82.1 ± 3.2 a	91.8 ± 1.1 a	80.8 ± 2.3 a	85.4 ± 2.1 a
T5	68.2 ± 0.7 b	64.1 ± 9.9 ab	72.6 ± 0.5 b	83.3 ± 0.9 b	68.3 ± 0.7 c	72.9 ± 2.1 b
T4	62.5 ± 0.5 b	56.5 ± 1.5 b	70.4 ± 1.7 b	82.0 ± 0.4 b	65.7 ± 0.9 c	71.9 ± 1.8 b
T2	47.1 ± 2.2 c	60.6 ± 5.3 b	54.2 ± 3.0 c	83.4 ± 1.3 b	48.3 ± 0.7 e	64.6 ± 3.8 bc
T1	51.3 ± 1.3 c	58.8 ± 1.9 b	53.6 ± 1.4 c	82.3 ± 0.5 b	55.5 ± 0.5 d	65.6 ± 3.1 bc

a *CK is the “Shepody” non-transgenic potato cultivar; the rest are transgenic potato lines. Each value ± standard error was obtained from three runs of moderate and severe stress treatments with three replicates in each run. Within a column, a higher coefficient indicates higher drought tolerance and the different letters denote significant differences at P < 0.05*.

Then, the six growth traits were integrated together to form an evaluation index using Equations (1–5) and to provide quantitative values in assessing the magnitude of the drought tolerance. The integrated evaluation indices (i.e., *D*-values, Table 2) were calculated by determining the relative weights of each of the six growth traits in influencing the drought tolerance. Subordinator functions were used to generate correlation coefficients ranging from 0.01 to 1.00, and a higher coefficient represents a greater contribution of the growth trait to the drought tolerance. These indices were used to rank the drought tolerance of the lines quantitatively. All the six transgenic lines had significantly higher *D*-values than the control (Table 2). The lines T6 and T7 had the *D*-values 8–9 times that of the control and 1.6–2.0 times that of the four other transgenic lines. These quantitative evaluations suggest that T6 and T7 have the greatest drought tolerance, followed by T5, T1, T2, and T4, and the control had the lowest drought tolerance.

**Table 2:** **Subordinator functional components and the integrated evaluation index for the transgenic potato lines overexpression of ***StPIP1*** and the check cultivar**.

**Genotype^a^**	**Subordinator functional Components^b^**						**Index^c^**	**Ranking**
	**μ(1)**	**μ(2)**	**μ(3)**	**μ(4)**	**μ(5)**	**μ(6)**		
CK	0.10	0.15	0.11	0.09	0.02	0.07	0.09	7
T7	0.95	0.62	0.70	0.95	0.81	0.90	0.70	2
T6	0.97	0.89	0.88	0.82	0.93	0.93	0.80	1
T5	0.63	0.46	0.63	0.49	0.70	0.53	0.49	3
T4	0.48	0.34	0.57	0.44	0.65	0.50	0.40	6
T2	0.07	0.40	0.14	0.49	0.32	0.27	0.41	5
T1	0.18	0.38	0.13	0.45	0.45	0.30	0.43	4
Weighted value	0.20	0.20	0.16	0.08	0.24	0.12		

a *CK is the “Shepody” non-transgenic potato cultivar; the rest are transgenic potato lines*.

b *The six subordinator functional components are (1) daily gain in plant height, (2)leaf area expansion rate, (3) stem thickness expansion rate, (4)relative water content,(5) tuber yield, and (6) survival rate after salt treatment*.

c *The integrated evaluation index was generated using the four subordinator functional components*.

Finally, a cluster analysis (cluster diagram not shown) was used to categorize the drought tolerance of the genetic materials into four categories: drought tolerance being very high (T6 and T7), high (T4 and T5), low (T1 and T2), and very low (the control). The persistence of the drought tolerance in the four groups was further verified in a controlled-environment experiment where the different levels of drought stresses (no stress, moderate stress, or severe stress) were imposed. At Day 45 in the stress treatments, the transgenic line T7 plants grew vigorously with more roots, while the control plants started wilting with fewer roots (Figure S8). At harvest, all plants produced tubers and no significant differences were shown under non-stress treatments (with normal irrigation). With the stress level increased from normal to moderate, the control plants decreased tuber yield by 37.8%, while the lines T7 and T6 plants decreased tuber yield by 5.5% (Table 3); With the drought stress further increased to severe level, the control plant decreased tuber yield by 97.4% while the lines T7 and T6 decreased by 39.7%.

**Table 3:** **Tuber yield of the non-transgenic control (CK) and the transgenic potato plants with high and very high expression of ***StPIP1*** gene under no-stress, moderate-and severe drought stress**.

**Group^a^**	**Tuber per plant (g)**		
	**Non-stress**	**Moderate drought**	**Severe drought**
CK	240.7 ± 1.5 a^b^	149.7 ± 2.0 d	6.3 ± 0.9 f
High	240.0 ± 1.0 a	195.2 ± 0.7 c	126.3 ± 1.7 e
Very High	241.2 ± 1.6 a	228.0 ± 4.1 b	145.5 ± 1.0 d

a *CK is the “Shepody” non-transgenic potato cultivar, the rest are transgenic potato lines with “High” (T1 and T5) and “Very high” (T6 and T7) degree of tolerance*.

b *Significant differences among means in each column according to Tukey's HSD test at P < 0.05 (n = 9, i.e., 3 replicates × 3 runs of the experiment)*.

With the stress level increased from normal to moderate, the control plants decreased root fresh weight by 67.6% and root dry weight by 40.0%; and with the drought stress furthered to the severe level, the control plants decreased root fresh weight by 69.8% and root dry weight by 80.0% (Table 4); by comparison, the values of the decreases for the lines T7 and T6 plants were 27.8 and 13.8%, respectively. The latter lines had significantly smaller decreases in root weights than the control plant. Similar trends of treatment effect on shoot fresh and dry weights were observed as the effects on the root traits: with the drought stress increased from normal to severe level, the control plant decreased shoot fresh weight by 82.5% and shoot dry weight by 62.9%, and the percent decreases were significantly smaller in the lines T7 and T6 plants. There were no differences in either fresh or dry weight of shoot or those of roots among the genetic lines under non-stress conditions.

**Table 4:** **Fresh and dry weight of potato of the non-transgenic control (CK) and the transgenic plants with high and very high expression of ***StPIP1*** gene under no-stress, moderate-and severe drought stress**.

**Weight (g)**	**Group^a^**	**Non-stress**	**Moderate drought**	**Severe drought**
Root fresh weight	CK	18.2 ± 0.7ab^b^	5.9 ± 1.1c	5.5 ± 2.1c
	High	25.4 ± 1.7a	18.5 ± 1.3ab	13.6 ± 1.9bc
	Very High	20.8 ± 1.4ab	24.5 ± 2.5a	15.0 ± 1.6b
Root dry weight	CK	2.5 ± 0.2bc	1.5 ± 0.3c	0.5 ± 0.0c
	High	3.3 ± 0.2b	2.7 ± 0.4b	2.1 ± 0.2bc
	Very High	2.9 ± 0.3b	4.5 ± 0.2a	2.5 ± 0.1bc
Shoot fresh weight	CK	187.0 ± 3.5bc	150.7 ± 4.0c	32.7 ± 4.3d
	High	239.8 ± 22.8bc	289.0 ± 33.2ab	160.9 ± 15.9c
	Very High	246.8 ± 18.9b	340.2 ± 2.2a	207.0 ± 8.9bc
Shoot dry weight	CK	44.7 ± 1.5bc	32.2 ± 1.6c	16.6 ± 2.2c
	High	72.1 ± 0.3a	72.0 ± 3.7a	40.9 ± 2.5bc
	Very High	67.8 ± 3.9ab	73.3 ± 7.7a	51.8 ± 4.2b

a *CK is the “Shepody” non-transgenic potato cultivar, the rest are transgenic potato lines with “High” (T1 and T5) and “Very high” (T6 and T7) degree of tolerance*.

b *Significant differences among means in each column according to Tukey's HSD test at P < 0.05 (n = 9, i.e., 3 replicates × 3 runs of the experiment)*.

### Photosynthetic responses to drought stress

**Figure 2:**
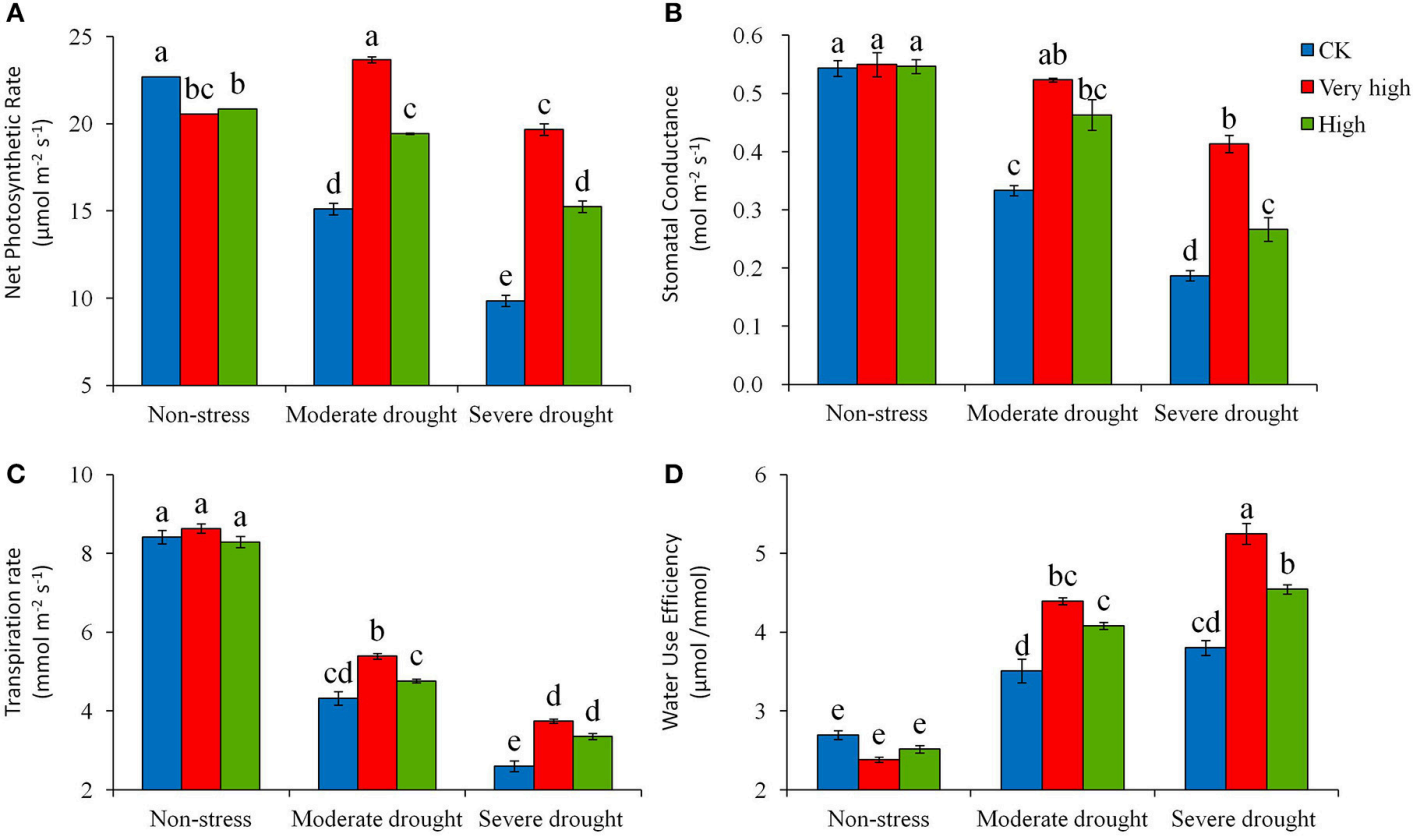
**Effects of different levels of drought stress on (A)** net photosynthetic rate, **(B)** stomatal conductance, **(C)** transpiration rate, and **(D)** instantaneous water use efficiency from gas exchange for the non-transgenic control (CK), and the transgenic lines with “Very high” (T6 and T7) and “High” (Tl and TS) expressions (as ranked in Table 2). Significant differences among means over the drought-stress treatments were determined according to Tukey's HSD test at *P* < 0.05. The line bars are standard errors (*n* = 9, i.e., 3 replicates × 3 runs of the experiment).

Drought stress affected a number of physiological traits in potato plants. Severe stress significantly decreased photosynthesis efficiency (Pn), stomatal conductance (Gs), and transpiration rate (Tr) in both transgenic and control plants. However, the magnitude of the decrease was significantly lower in the transgenic lines than in the control (Figure 2). Measured Day 30 in stress treatment, the transgenic lines in the “Very high” category decreased Pn slightly under the severe drought stress, while the control plant decreased Pn by 33% under moderate stress and 56% under severe stress (Figure 2A); a similar trend of response to drought stress was found in Gs and Tr, where the control plants decreased Gs by 39% under moderate stress and 65% under severe stress (Figure 2B); with the values of Tr decreases being 49 and 69%, respectively (Figure 2C). These defending responses of the transgenic lines to drought stress enhanced water use efficiency (WUE), as evidenced that under severe stress the transgenic lines increased WUE more prominently than the check (Figure 2D).

### Antioxidant enzyme activities and biochemical responses

**Figure 3:**
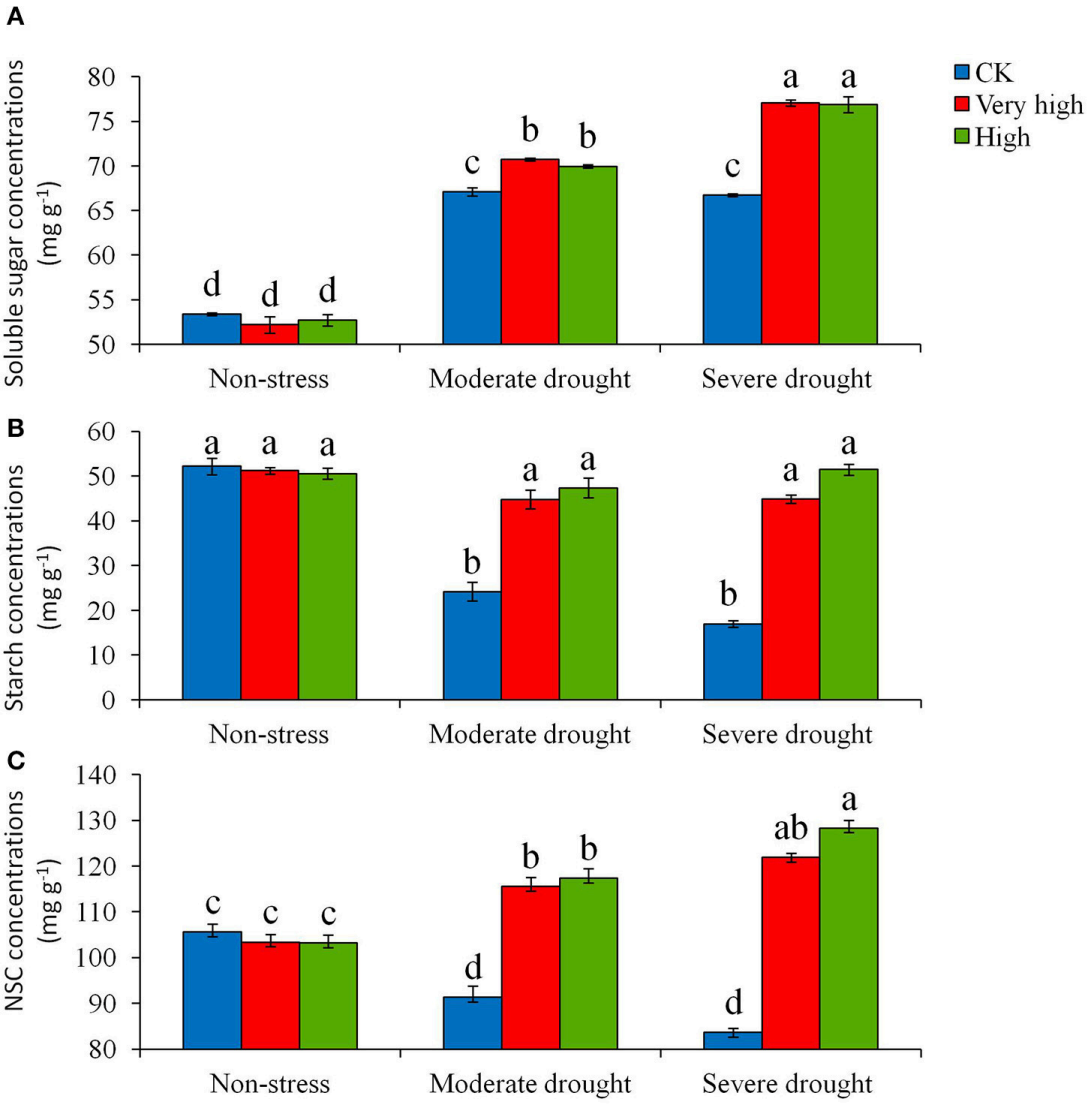
**Effects of various levels of drought stress on (A)** soluble sugar, **(B)** starch and **(C)** total NSC concentrations for the non-transgenic control (CK), and the transgenic lines with “Very high” (T6 and T7) and “High” (Tl and TS) expressions (as ranked in Table 2). Significant differences among means over the drought-stress treatments were determined according to Tukey's HSD test at *P* < 0.05. The line bars are standard errors (*n* = 9, i.e., 3 replicates × 3 runs of the experiment).

The soluble sugar, starch, total nonstructural carbohydrates (NSC) concentrations, superoxide dismutase (SOD), peroxidase (POD), catalase (CAT) in the leaves of young potato plants with overexpression of *StPIP1* gene were measured at each of the three stress levels. Significant (*P* < 0.05) differences in soluble sugar concentration were detected among the different lines and among treatments (Figure 3A). The transgenic lines increased soluble sugar concentration remarkably compared to the control under moderate and severe drought stress treatments. The plants in the “Very high” and “High” categories had the similar responses to the stress. In contrast, the control plants had the lowest sugar and starch concentrations (Figure 3B). Also, the responses of total NSC to drought stress varied significantly among the lines (Figure 3C). Overall, the control plants had the lowest total NSC concentration under drought stress. With severe stress, total NSC concentration in the transgenic lines was 1.5 times higher than that in the control under (Figure 3C).

**Figure 4:**
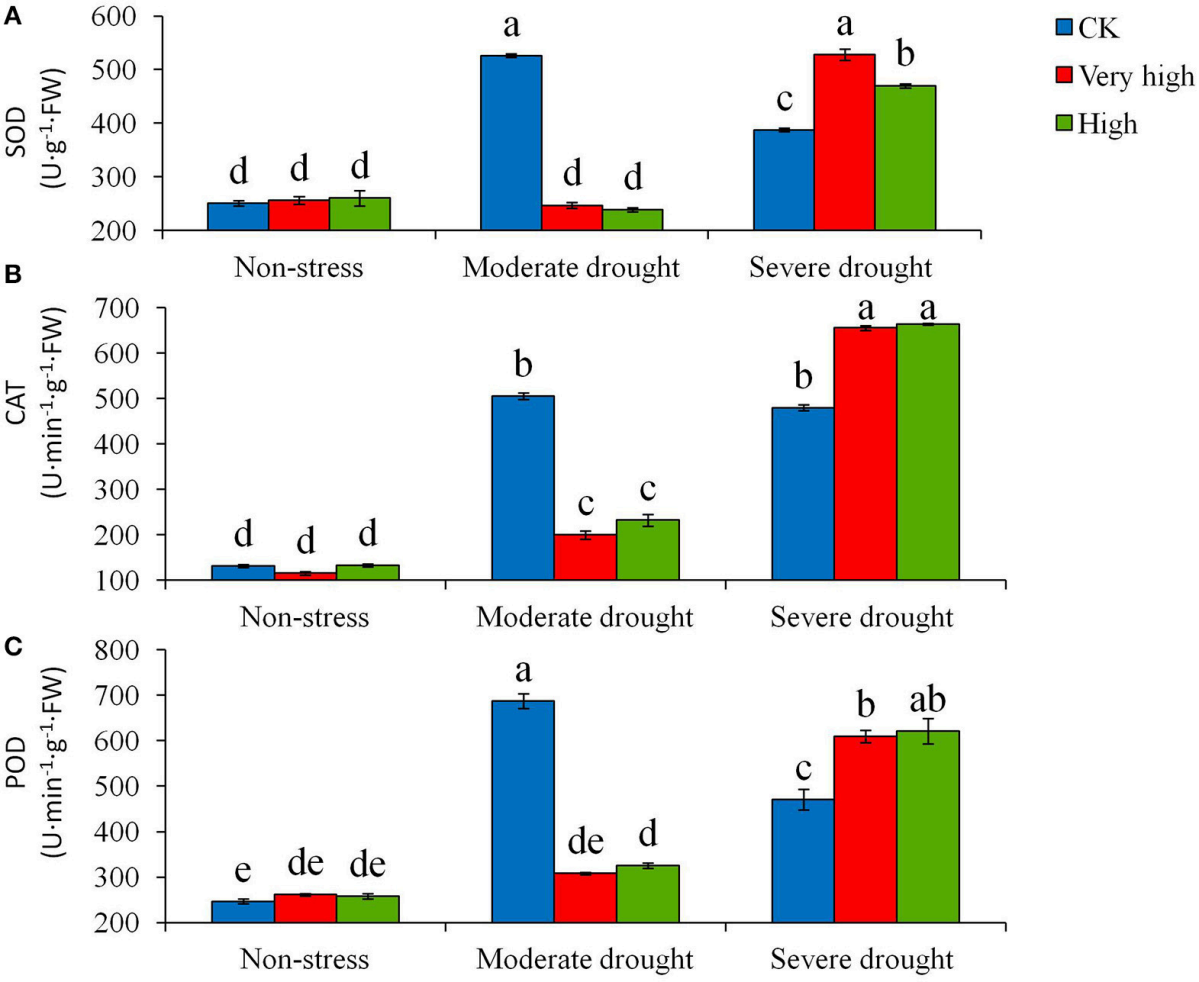
**Effects of different levels of drought stress on (A)** superoxide dismutase (SOD), **(B)** catalase (CAT) and **(C)** peroxidase (POD) for the non-transgenic control (CK), and the transgenic lines with “Very high” (T6 and T7) and “High” (Tl and TS) expressions (as ranked in Table 2). Significant differences among means over the drought-stress treatments were determined according to Tukey's HSD test at *P* < 0.05. The line bars are standard errors (*n* = 9, i.e., 3 replicates × 3 runs of the experiment).

Furthermore, the biochemical analysis showed that the drought stress treatments affected the activity of many anti-oxidative enzymes, such as SOD, POD, and CAT. Moderate and severe drought stresses stimulated enzymatic activities in general, but the magnitude of the responses differed between the transgenic and control plants (Figure 4). In particular, under severe stress, the transgenic lines increased the enzymatic activities significantly (*P* < 0.05) whereas the control plants had a low enzymatic activity.

### Physiological responses to drought stress

**Figure 5:**
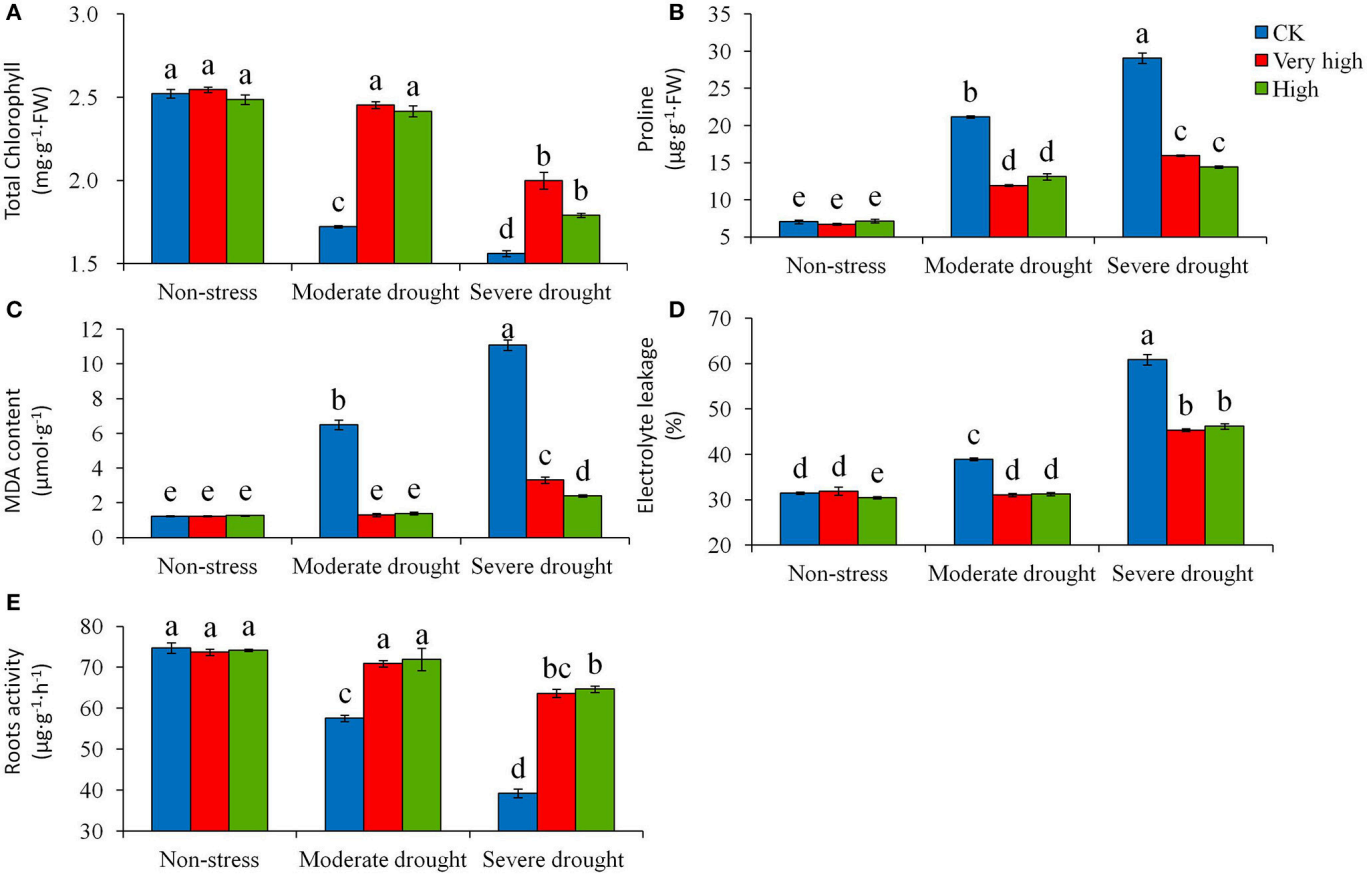
**Effects of various levels of drought stress on (A)** total chlorophyll content, **(B)** proline content, **(C)** malondialdehyde (MDA) content, **(D)** electrolyte leakage, and **(E)** roots activity for the non-transgenic control (CK), and the transgenic lines with “Very high” (T6 and T7) and “High” (Tl and TS) expressions (as ranked in Table 2). Significant differences among means over the drought-stress treatments were determined according to Tukey's HSD test at *P* < 0.05. The line bars are standard errors (*n* = 9, i.e., 3 replicates × 3 runs of the experiment).

Total chlorophyll content decreased significantly (*P* < 0.05) with the increase of drought stress levels for all the genotypes evaluated in the study (Figure 5A). The magnitude of the decrease in chlorophyll content varied largely, with the transgenic plants in the “Very high” and “High” categories decreasing significantly less than the control. By day 30 of stress treatments, the control plants lowered chlorophyll content from 2.5 mg g^−1^ of FW at no-stress to 1.72 mg g^−1^ at moderate stress, and furthered to 1.56 mg g^−1^ at severe stress, representing a decrease of 32 and 38%, respectively. On average, total chlorophyll contents of the transgenic lines in the “Very high” and “High” groups were 25% higher than in the control. The reverse trend of effect was found for proline content: with drought stress the control plants increased proline content significantly more than the transgenic lines (Figure 5B). By Day 30 of severe stress treatment, proline content in the control plant reached a level of 29.1 μg^−1^ g^−1^ of FW, which was about 2 times the average of the transgenic lines. The MDA content (Figure 5C) followed a similar trend of effect as the proline content. The magnitude of the increases in MDA varied among the genotypes. Treated for 30 days under drought stress, the control plants increased MDA content from 1.23 mg g^−1^ of FW at no-stress to 6.50 at moderate stress and furthered to 11.09 at severe stress, representing an increase of 5.3 and 9.0 times, respectively. The transgenic lines increased a least amount of MDA. In contrast to the variables described above, the response of electrolyte leakage to drought stress varied substantially among genotypes (Figure 5D). A similar level of electrolyte leakage was found in the transgenic lines among the three levels of drought stress, but electrolyte leakage in the control plant increased significantly both in the moderate and severe stresses (Figure 5D).

The root activity assay showed that the continuous drought challenges decreased root activity for all genotypes evaluated, with the control plant decreasing the root activity more substantially (*P* < 0.05) than the transgenic lines in the moderate and severe drought stress. At the high stress levels, the roots activity of the control plants was about half of the transgenic lines (Figure 5E). The over-expression of *StPIP1* gene effectively improved the root activity of potato plants.

## Discussion

Plant aquaporins are responsible for water transmembrane transport, which plays an important role in alleviating drought stress (Sreedharan et al., 2013; Moshelion et al., 2015). Most of the AQP-related research in the literature focus on gene cloning and expression analysis (Wu et al., 2009; Hu et al., 2012; Yin et al., 2015), and there are fewer reports determining the relationship of AQPs in alleviating carbon starvation due to drought in potato. Our research team has identified a plasma membrane intrinsic protein of *StPIP1* gene and cloned from the leaf of potato (Wu et al., 2009). The plasmalemma gene has then been successfully transferred into a most abundantly-grown potato cultivar. The expression of the *StPIP1* gene was verified using various molecular tools, including PCR-Southern blot, RT-PCR, and QRT-PCR. This gene contains an unabridged open reading frame of 867 bp, encoded 288 amino acids. Our study demonstrated the strong roles the *StPIP1* gene plays in alleviating drought tolerance, reflected by various biophysicochemical properties of the transgenic lines, including antioxidant enzyme activities, nonstructural carbohydrates, photosynthesis efficiency, stomatal conductance, and transpiration rate.

In the present study, the expression of *StPIP1* gene was assessed in the different plant parts and at the different growth stages, which gives a comprehensive understanding how *PIP1* gene transcription in different plant tissues may affect potato tolerance to drought. The expression of *StPIP1* gene was found in leaves, stems and roots, and it was the roots where the gene expression was most abundant. The roots with a highest *StPIP1* expression exhibited greatest root activity, suggesting that roots are the most favorable loci for *StPIP1* gene expression in potato. Also, large differences of *StPIP1* gene expression were found among the transgenic lines, with the lines T6 and T7 having 5–15 times the expression of the non-transgenic control plant and 2–15 times that of the other transgenic lines. These results suggest that gene transformation may provide opportunity for the genetic enhancement of potato to drought tolerance, while the gene expression and the associated biophysicochemical traits must be evaluated thoroughly for their persistence. Additionally, we found that the relative expression of the *StPIP1* gene was affected by the length of stress treatment, with the highest expression occurring 48 h after mannitol osmotic stress treatment. These results indicate that the regulatory mechanism of endogenous aquaporins in response to drought stress is complex in potato.

In the recent years, many attempts have been made to understand the role of aquaporins in enhancing drought tolerance in plants, and results have been inconsistent, varying with growing conditions and crop species. For example, the overexpression of *Arabidopsis PIP1b* in transgenic tobacco was found to improve plant vigor under favorable conditions but no effect was shown under drought-stressed conditions (Aharon, 2003). The overexpression of the PIP1 subgroup gene *TaAQP8* was found to improve drought tolerance in transgenic tobacco (Hu et al., 2012), whereas in banana (*Musa acuminate* L.), the *PIP1* subfamily AQP gene *MaPIP1;1* was found to be associated with decreased membrane injury and improved osmotic adjustment (Xu et al., 2014). However, little has been reported how the *PIP* overexpression in potato might be related to biophysicochemical properties of the plant under drought. In a study with *Solanaceae* plants, StPIP1 protein was found to share a high homology with PIPs, such as NtAQP1, PhPIP, and CaPIP1 (Yin et al., 2015), but the mechanism underlying the effects of these proteins on the improvement of drought tolerance is poorly understood.

In many published studies, researchers attempt to evaluate the degree of drought tolerance using selected plant-growth variables, and often the evaluation with selected traits provide a biased ranking of tolerance. In the present study, we developed an evaluation index, where several key performance traits of the plants were integrated together to give a quantitative ranking of the tolerance to drought stress. Two of the seven transgenic lines (i.e., T7 and T6) had the highest drought tolerance; they were 8.5 times that of the non-transgenic control and 1.8 times that of the other transgenic lines. The two transgenic lines with highest tolerance index ranking also had the greatest *StPIP1* gene expression, indicating that the plasmalemma aquaporin encoding gene *StPIP1* plays an important role for promoting the plant growth under stress and thus enhancing the level of tolerance to the drought stress.

Carbon starvation and hydraulic failure have been considered the two main mechanisms responsible for plant death under severe drought (Salmon et al., 2015). Carbon starvation may occur with the depletion of non-structural carbohydrates (NSC) in response to stomatal closure and reduced carbon assimilation during extended drought, whereas hydraulic failure may occur with xylem dysfunction from runaway embolism (Gentine et al., 2016). These two processes may occur simultaneously in plant in response to drought, and the avoidance of drought-induced hydraulic failure via stomatal closure may lead to carbon starvation and a cascade of downstream effects (McDowell et al., 2008). Also, the process of carbon starvation may cause a reduction in plant photosynthesis and yield. A number of researchers have reported that NSC is one of the important indicators of carbon starvation as it reflects the degree of carbon starvation under drought stress (Rosas et al., 2013; Zhang et al., 2015b). In the present study, we measured NSC in the plants with *StPIP1* expression vs. those without at the various growth stages. Our results showed that the total NSC concentration was significantly higher in the transgenic lines with higher *StPIP1* expression than in the control plants. These observations demonstrated that the transgenic plants did not experience carbon starvation under severe drought stress but the control (non-transgenic) plants did. These results suggest that the constitutive expression of *StPIP1* gene in the transgenic potato help maintain the whole-plant carbon level under drought.

Whether the ability of retaining the non-starving carbon level in the transgenic lines is a direct or an indict effect of the *StPIP1* expression was undefined in the present study. More in-depth research is required to elucidate the effect. However, *StPIP1* expression seems to offer multiple functions, as evidenced by the fact that *StPIP1* expression promoted the activities of antioxidant enzymes SOD, CAT, and POD, increased total chlorophyll content and decreased MDA content and electrolyte leakage destruction, and reduced cell damage under drought. Physiologically, the expression of *StPIP1* gene had several positive outcomes, such as maintaining the net photosynthesis, balancing stomatal conductance and transpiration, and enhancing root activity. Consequently, the plants with the expression of *StPIP1* minimized biomass and yield loss and improved water use efficiency, under moderate to severe drought stresses.

## Conclusion

This study tested the hypothesis that over expression of *StPIP1* in potato improves plant water balance under drought stress thereby improving carbon assimilation and storage. Our results showed that under prolonged dehydration stress, the transgenic plants with a high expression of *StPIP1* accumulated more NSC in plant tissues, increased the ability to maintain transpiration, and promoted various biophysicochemical activities that help minimize the drought-induced damage. The improved physiochemical activities ensure continuous CO_2_ uptake and nutrient supplies, alleviating the deleterious effects of drought stress. The constitution of *StPIP1* in potato plants improved water status under drought that promoted a conversion from carbon starvation to carbon non-starvation. An overexpression of the *StPIP1* extended the ability to maintain plant growth and yield even under severe stress conditions.

## Author contributions

LW and JZ conceived the research and designed all experiments; LW, YL, SF, DL, BY, and HY collected molecular and greenhouse data; LW analyzed the data with help from YL, SF, and JY; LW wrote the paper with the assistance of JZ and JY; LW, JZ, YL, and JY revised and approved the final manuscript.

### Conflict of interest statement

The authors declare that the research was conducted in the absence of any commercial or financial relationships that could be construed as a potential conflict of interest.

## Abbreviations

FW: Fresh weight
NSC: nonstructural carbohydrates
RWC: relative water content
MDA: malondialdehyde
SOD: superoxide dismutase
CAT: catalase
and POD: peroxidase
Pn: Net photosynthetic rate
WUE: water use efficiency
Gs: Stomatal conductance
Tr: Transpiration rate.
