# Supplementary figures and images for: Roles of Plasmalemma Aquaporin Gene StPIP1 in Enhancing Drought Tolerance in Potato
Source: Front Plant Sci. 2017 Apr 25;8:616. doi: 10.3389/fpls.2017.00616 (PMC5403905; doi:10.3389/fpls.2017.00616)

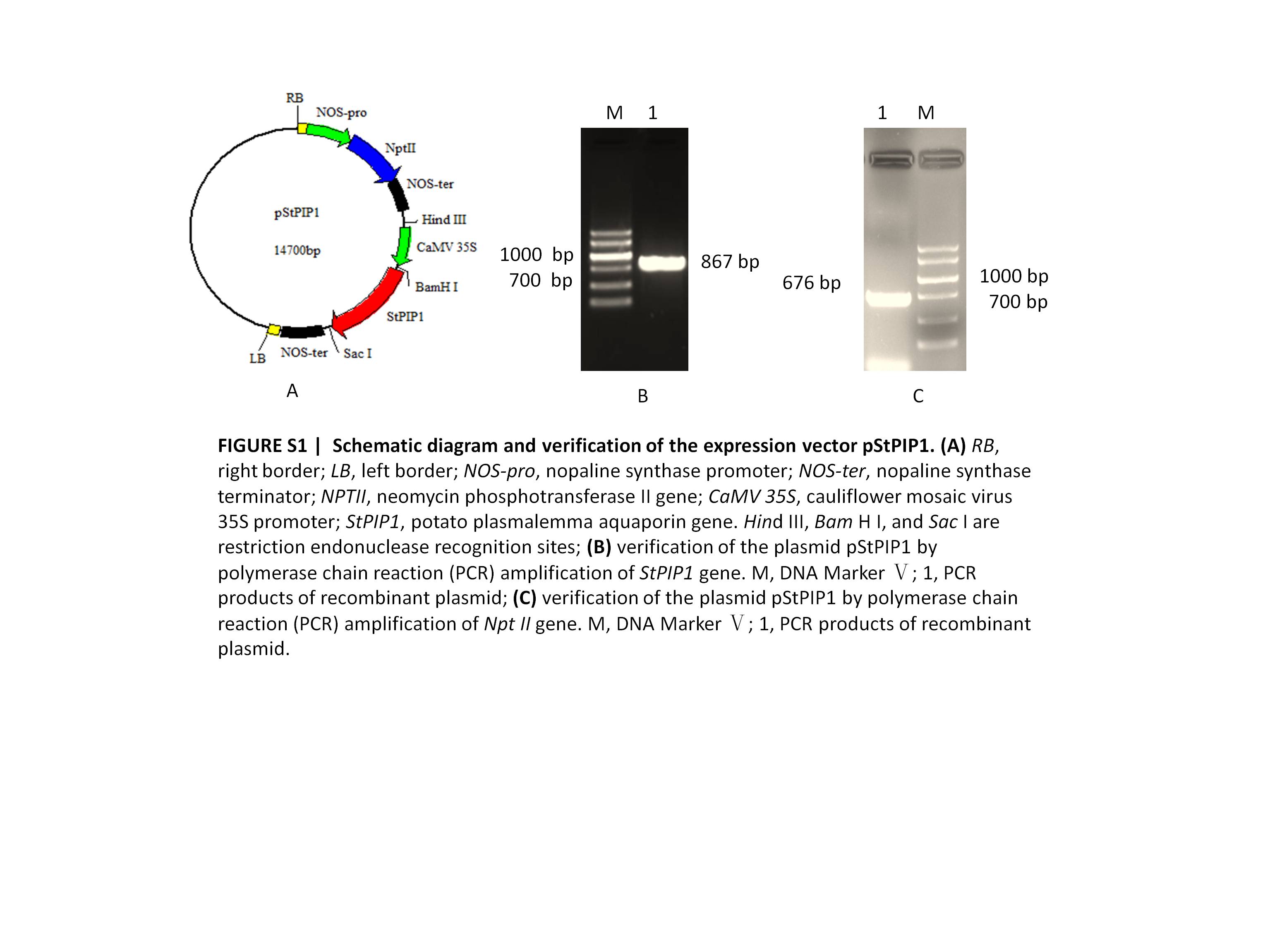

Supplement: Supplementary file 1 [file Image1.JPEG]

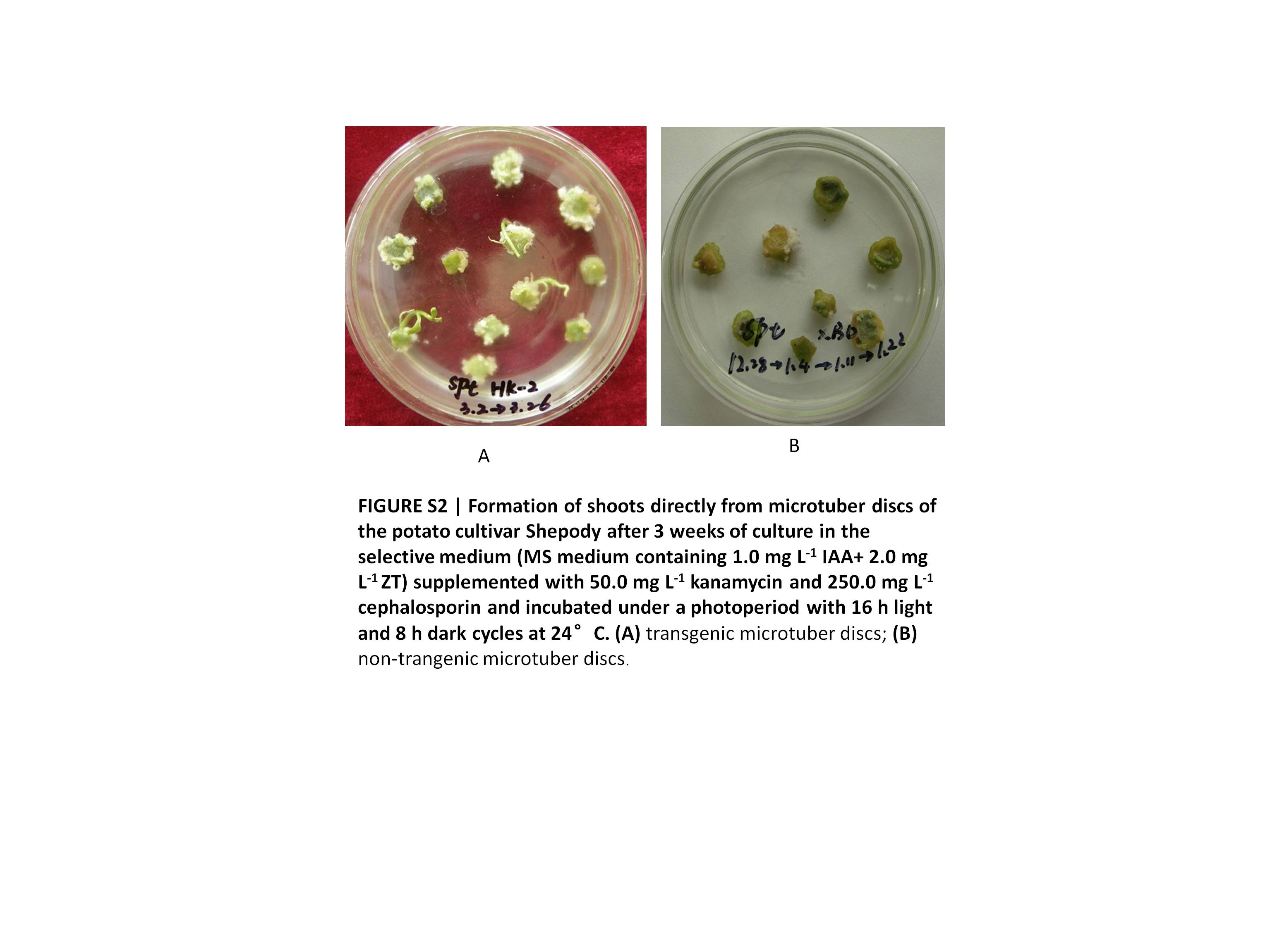

Supplement: Supplementary file 2 [file Image2.JPEG]

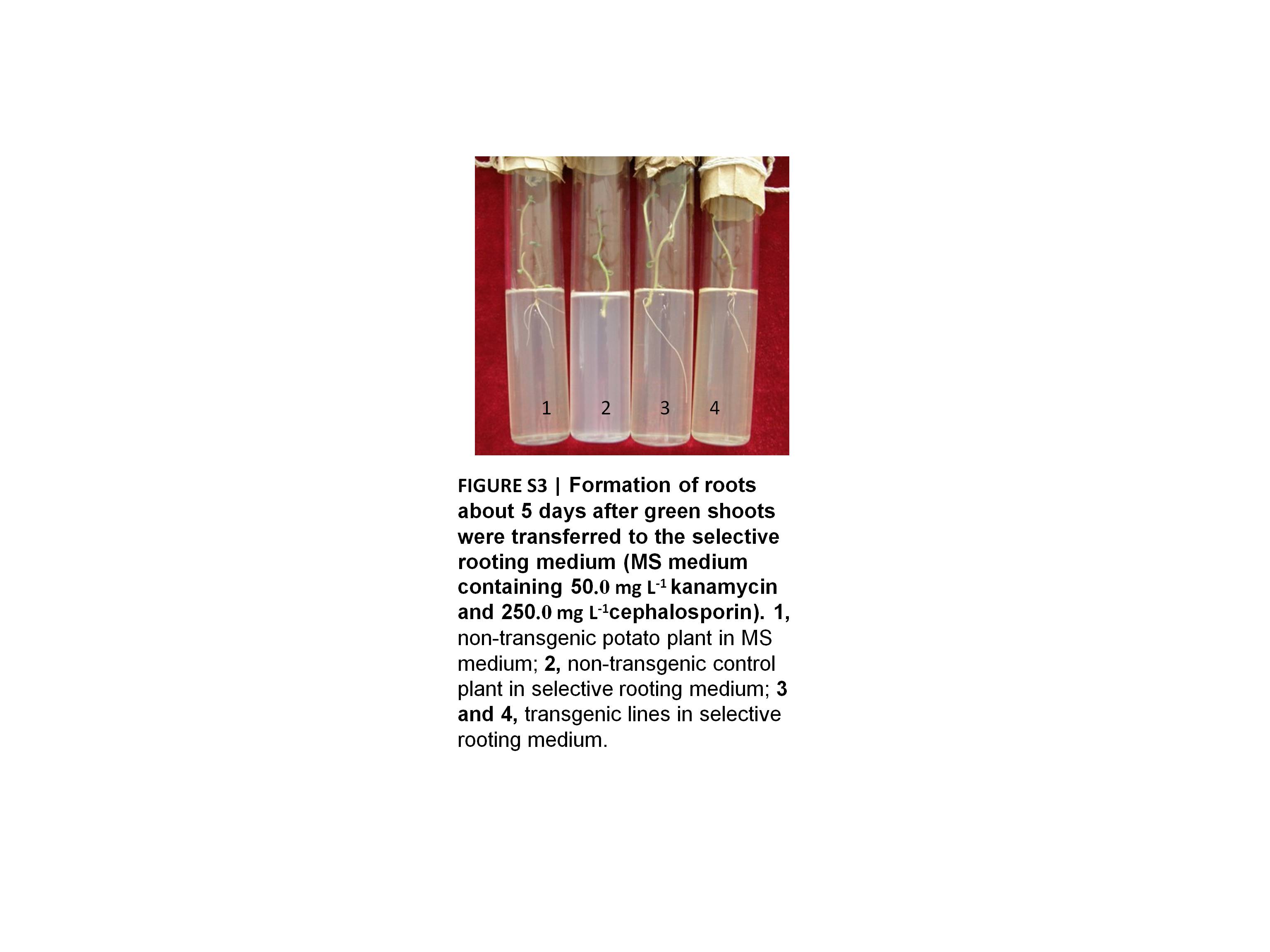

Supplement: Supplementary file 3 [file Image3.JPEG]

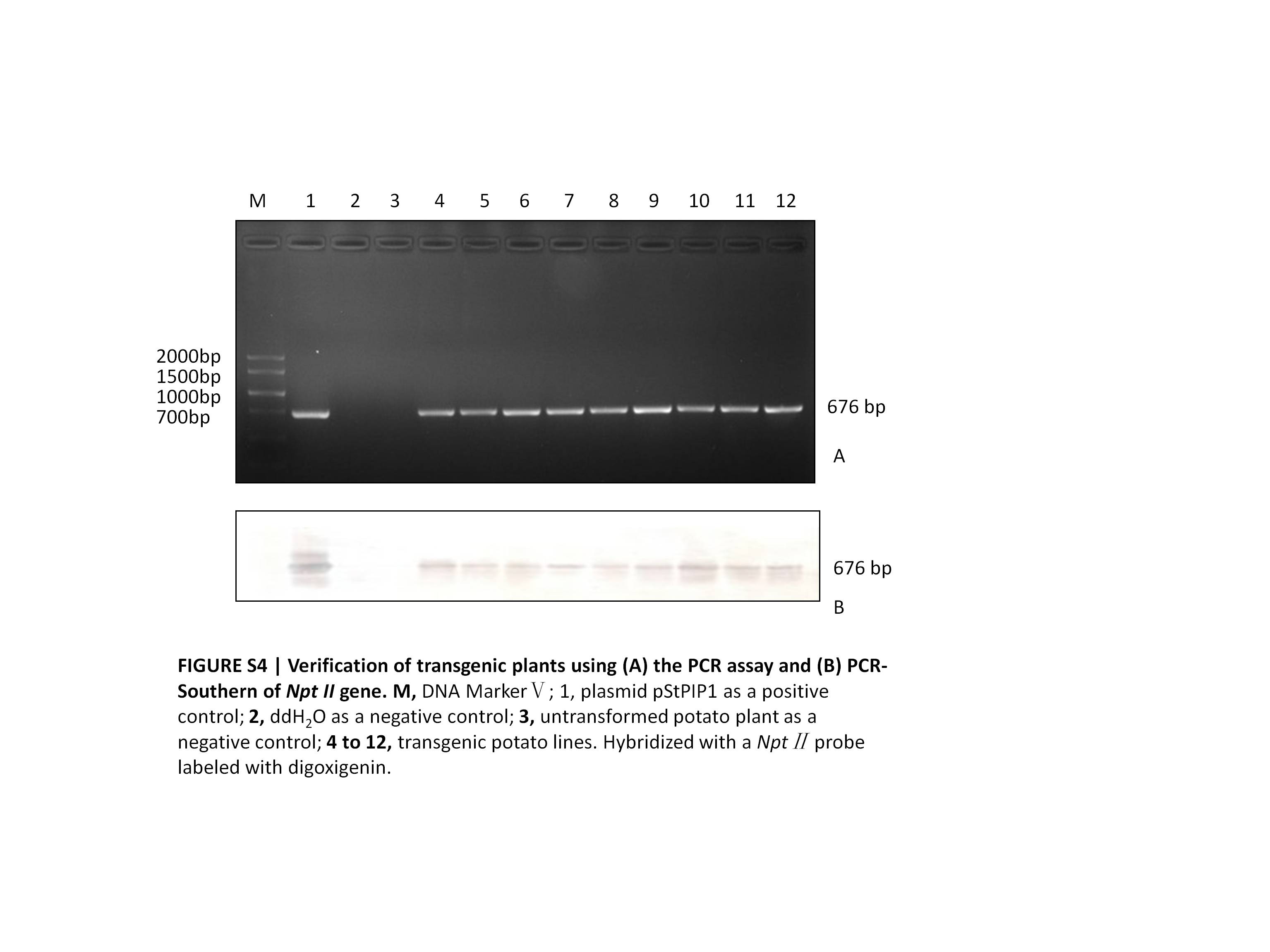

Supplement: Supplementary file 4 [file Image4.JPEG]

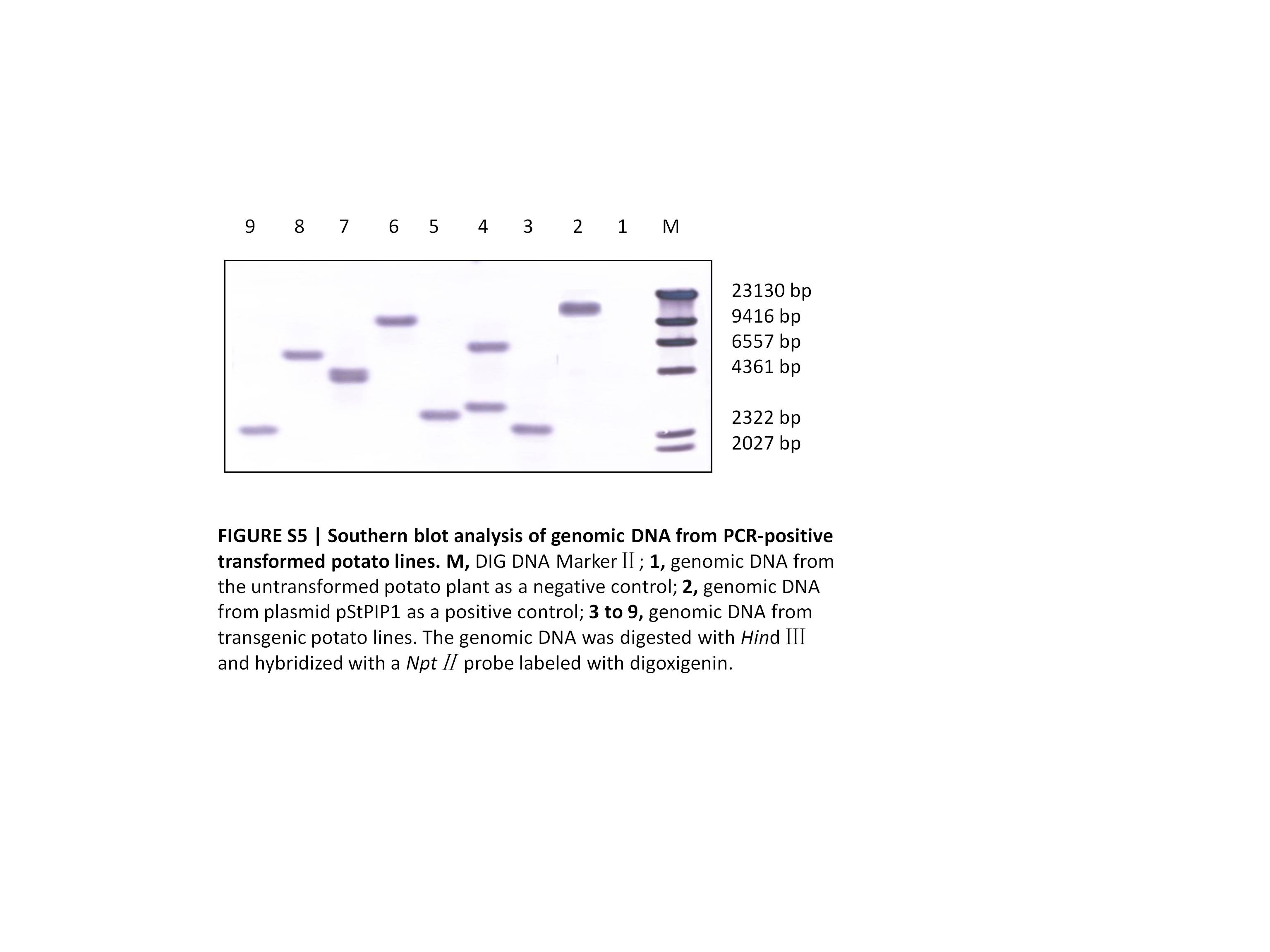

Supplement: Supplementary file 5 [file Image5.JPEG]

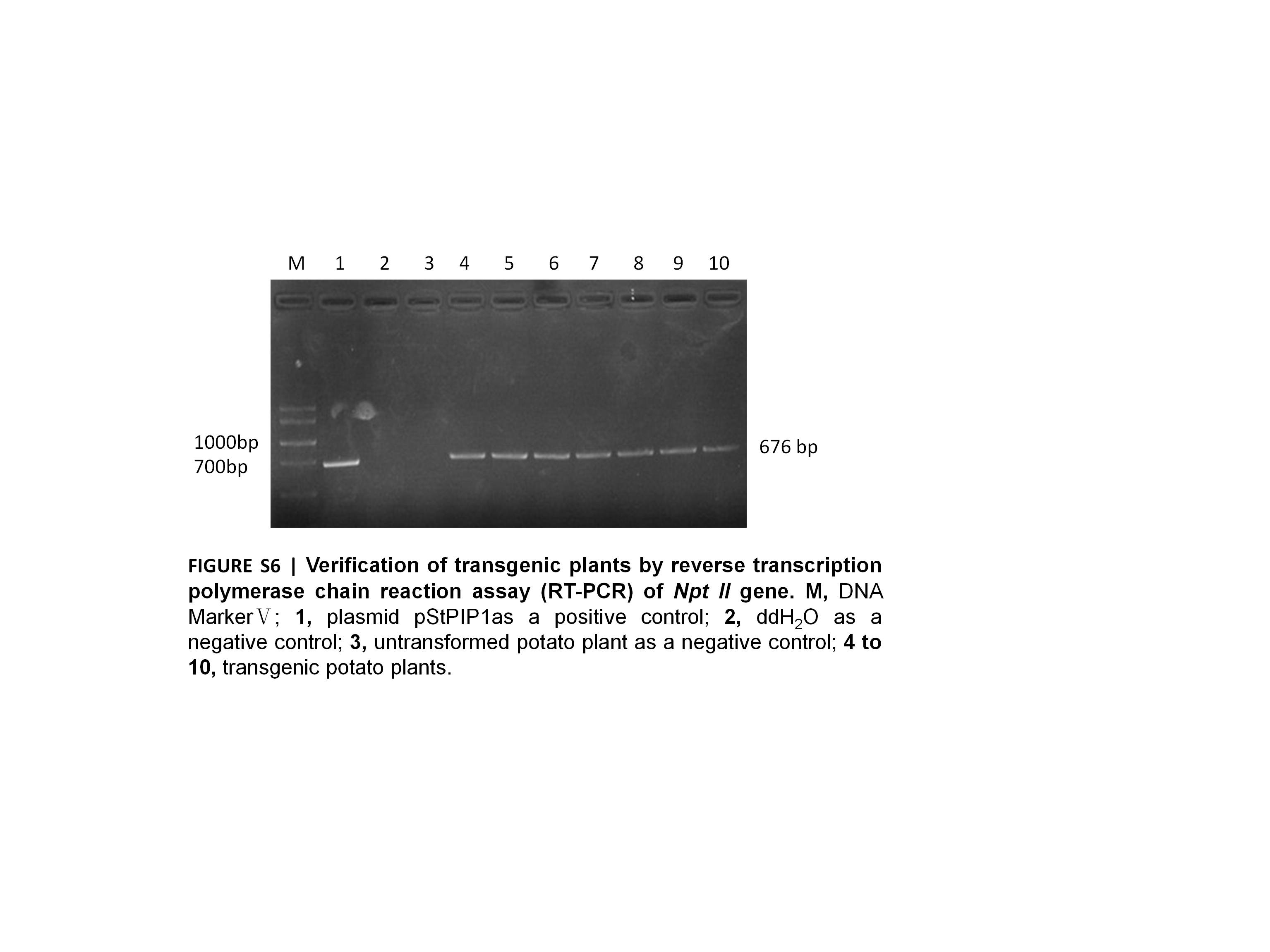

Supplement: Supplementary file 6 [file Image6.JPEG]

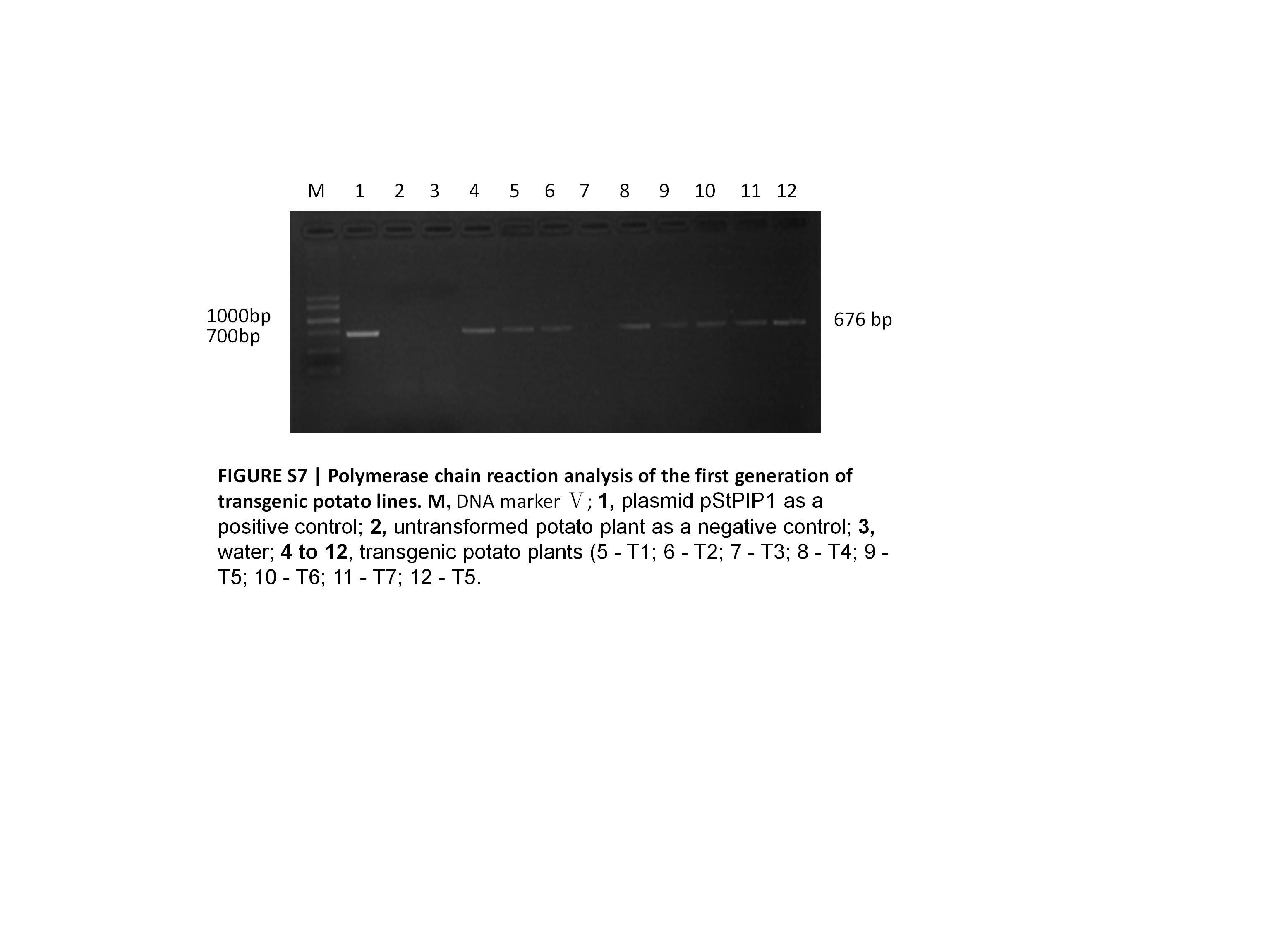

Supplement: Supplementary file 7 [file Image7.JPEG]

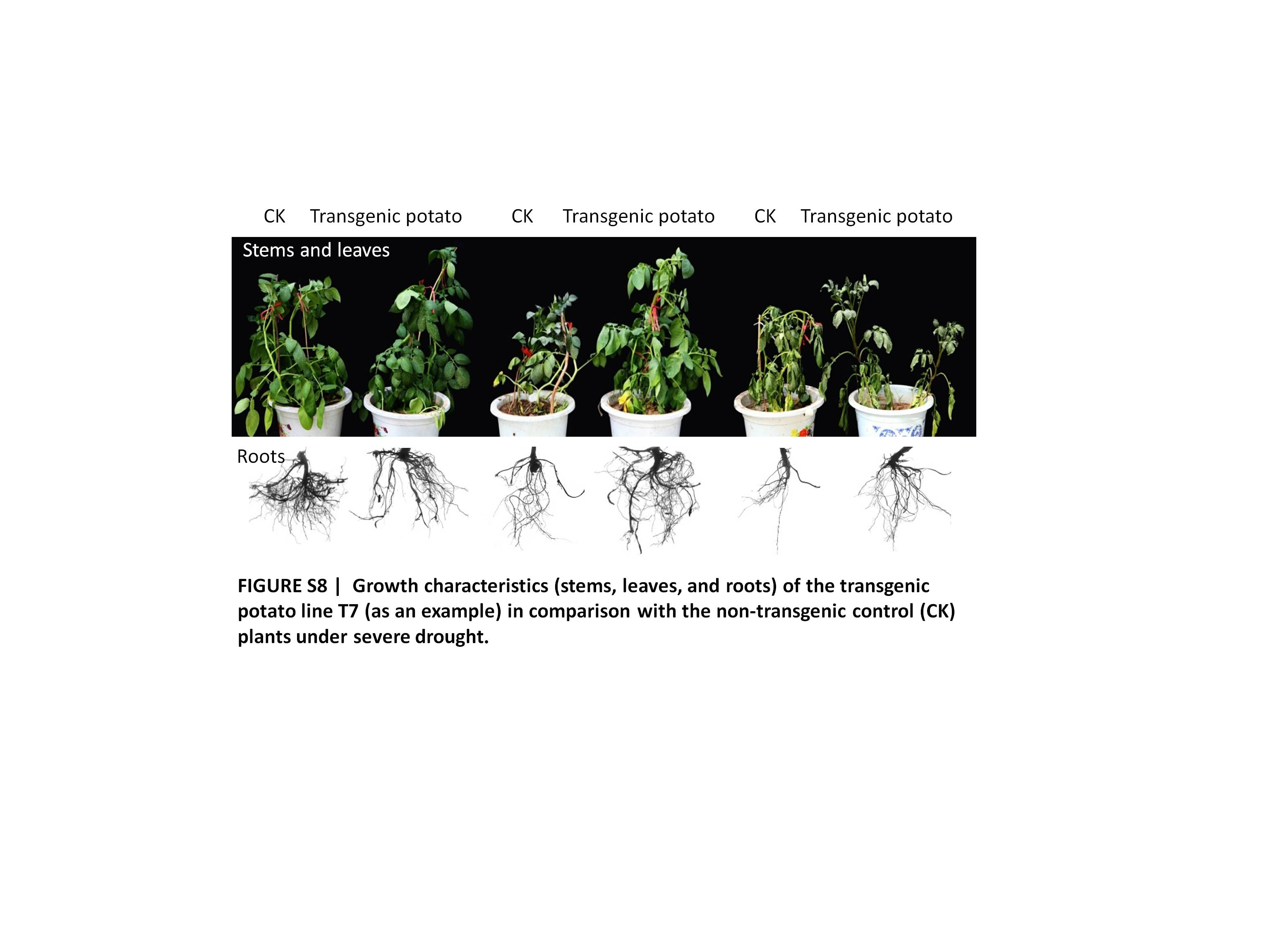

Supplement: Supplementary file 8 [file Image8.JPEG]
